# A novel simple risk model to predict the prognosis of patients with paraquat poisoning

**DOI:** 10.1038/s41598-020-80371-5

**Published:** 2021-01-08

**Authors:** Yanxia Gao, Liwen Liu, Tiegang Li, Ding Yuan, Yibo Wang, Zhigao Xu, Linlin Hou, Yan Zhang, Guoyu Duan, Changhua Sun, Lu Che, Sujuan Li, Pei Sun, Yi Li, Zhigang Ren

**Affiliations:** 1 Emergency Department, The First Affiliated Hospital of Zhengzhou University, Zhengzhou, 450052 China; 2 Department of Infectious Diseases, The First Affiliated Hospital of Zhengzhou University, Zhengzhou, 450052 China; 3 Gene Hospital of Henan Province, Precision Medicine Center, The First Affiliated Hospital of Zhengzhou University, Zhengzhou, 450052 China; 4 Department of Emergency Medicine, Shengjing Hospital of China Medical University, Shenyang, 110001 China; 5 Emergency Department, Chinese Academy of Medical Sciences, Peking Union Medical College Hospital, Beijing, 100730 China

**Keywords:** Medical research, Risk factors

## Abstract

To identify risk factors and develop a simple model to predict early prognosis of acute paraquat (PQ) poisoning patients, we performed a retrospective cohort study of acute PQ poisoning patients (n = 1199). Patients (n = 913) with PQ poisoning from 2011 to 2018 were randomly divided into training (n = 609) and test (n = 304) samples. Another two independent cohorts were used as validation samples for a different time (n = 207) and site (n = 79). Risk factors were identified using a logistic model with Markov Chain Monte Carlo (MCMC) simulation and further evaluated using a latent class analysis. The prediction score was developed based on the training sample and was evaluated using the testing and validation samples. Eight factors, including age, ingestion volume, creatine kinase-MB [CK-MB], platelet [PLT], white blood cell [WBC], neutrophil counts [N], gamma-glutamyl transferase [GGT], and serum creatinine [Cr] were identified as independent risk indicators of in-hospital death events. The risk model had C statistics of 0.895 (95% CI 0.855–0.928), 0.891 (95% CI 0.848–0.932), and 0.829 (95% CI 0.455–1.000), and predictive ranges of 4.6–98.2%, 2.3–94.9%, and 0–12.5% for the test, validation_time, and validation_site samples, respectively. In the training sample, the risk model classified 18.4%, 59.9%, and 21.7% of patients into the high-, average-, and low-risk groups, with corresponding probabilities of 0.985, 0.365, and 0.03 for in-hospital death events. We developed and evaluated a simple risk model to predict the prognosis of patients with acute PQ poisoning. This risk scoring system could be helpful for identifying high-risk patients and reducing mortality due to PQ poisoning.

## Introduction

The non-selective contact herbicide paraquat (PQ) is predominantly used in developing agricultural countries^1,2^. PQ is lethal when ingested orally, and there are still no effective and specific antidotes. Patients with acute PQ poisoning usually die within several days to weeks after exposure due to hypoxemia or multiple organ failure. PQ poisoning results in increased medical resource use, causing a considerable economic impact^3–6^. Therefore, timely clinical outcome evaluation and risk assessment for critically ill PQ poisoning patients and essential for appropriate medical resource allocation, which has become an important public health and social security agency issue concerning doctors and patients.

To our knowledge, several systems have been reported to predict the prognosis of patients with PQ poisoning, including the Acute Physiology and Chronic Health Evaluation II (APACHE II) score^7^, Sequential Organ Failure Assessment (SOFA) score^8^, the Severity Index of PQ Poisoning (SIPP)^9^, Poisoning Severity Score (PSS)^10^, and several equations and nomograms based on large cohort studies^11–13^. Most of these models are suitable for critically ill patients rather than patients with minimal exposure or early-stage patients with mild symptoms. Moreover, these scoring systems can fail to predict mortality and conduct risk assessment for PQ poisoning patients instantly because of their complicated calculations or the unavailability of laboratory tests. Thus, establishing an effective, simple, and universal predictive model based on common laboratory tests would be invaluable for risk stratification and therapeutic regimen adjustment for patients with acute PQ poisoning of all stages.

Here, based on data from 1199 patients with PQ poisoning from two large academic hospitals in China, we developed and evaluated a simple risk model by identifying significant clinical risk factors to predict in-hospital death. Data for our study were collected from the medical records of acute PQ poisoning patients from different time periods and geographic regions. This study describes a well-designed and easy to administer tool for predicting in-hospital death of PQ poisoning patients using a combination of simple and clinically relevant variables.

## Methods

### Study samples and data sources

**Figure 1:**
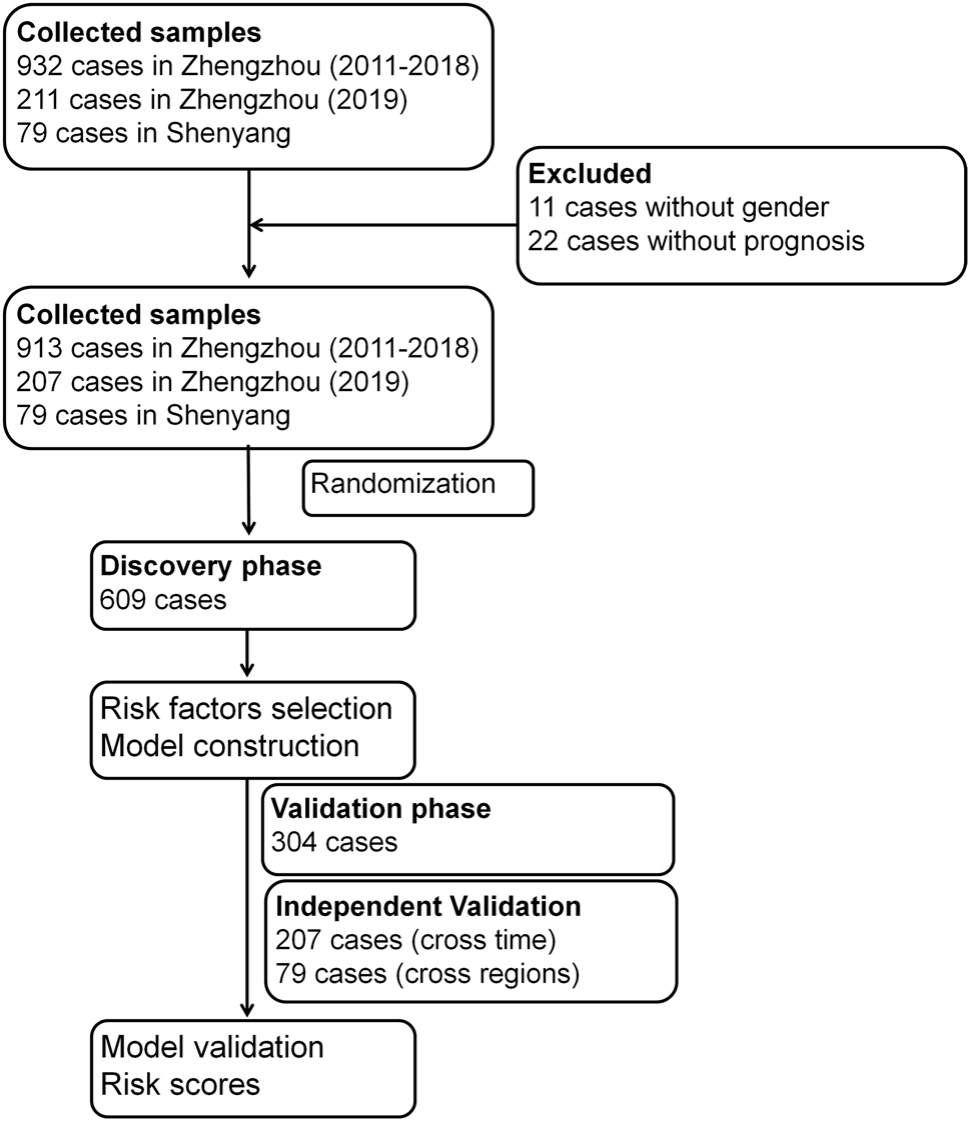
Study design. A total of 932 patients were enrolled during January and February 2019. An additional 207 patients from Zhengzhou 2019 and 79 from Shenyang Shengjing hospital were included as the external validation data sets. Patients without sex (n = 11) and prognosis (n = 22) information were excluded. Then, 913 unique patients were randomly divided into two mutually exclusive samples for training (66.7% [609 patients]) and testing (33.3% [304 patients]). All analyses were conducted using SAS^®^ statistical software version 9.4 (https://www.sas.com).

The PQ poisoning study included 932 patients from January 1, 2011 to December 31, 2018. Patient information was collected during January and February of 2019. An additional 207 cases from Zhengzhou 2019 and 79 from Shenyang Shengjing hospital were collected as external validation samples. Patients with missing information on sex (n = 11) and prognosis (n = 22) were excluded. The final sample included 913 unique patients who were randomly divided into two mutually exclusive training (66.7% [609 patients]) and test (33.3% [304 patients]) sets. The training sample was used to select risk factors, and the test sample was used for evaluation. Cases from Zhengzhou 2019 (n = 207) were used as a validation sample from a different time period and 79 cases from Shenyang as a validation sample from a different hospital (Fig. 1).

### Ethical statement

This study was approved by the Institutional Review Board of the First Affiliated Hospital of Zhengzhou University (2017-XY-002), and all experimental protocols of the study were approved by the ethics committee. Written informed consent was obtained from all enrolled patients. This study was conducted in accordance with the ethical standards of the Declaration of Helsinki 1975.

### Potential risk factors and outcome

We selected candidate risk factors that were clinically meaningful, reliable, and easily collected, which occurred with a frequency of more than 1%. Initial factors included patient demographic characteristics (age, sex), PQ ingestion time and volume, and laboratory test results (blood urea nitrogen [BUN], creatine kinase [CK], CK-MB, gamma-glutamyl transferase [GGT], mean platelet volume [MPV], procalcitonin [PCT], platelet [PLT], white blood cell [WBC], and neutrophil [N] counts, aspartate aminotransferase [AST], alanine aminotransferase [ALT], and creatinine [Cr]). Detailed information on these variables in the 1199 samples is shown in “Supplementary file S1”, Table S1. To facilitate the risk score calculation, we examined the nonlinear relationship of each continuous factor with the outcome and categorized it using a cut-off point while taking into account both in-hospital death rates and sample sizes (“Supplementary file S1”, Fig S1). Parameters with missing data rate > 15% were excluded. Factors with missing values were imputed using ten imputations. The final imputed value was the average of the ten imputations. Missing values ranged from 1.1% (age) to 13.3% (CK-MB). The primary outcome was in-hospital death, which defined as death during hospitalization.

### Statistical analysis

#### Risk factor selection and evaluation

Using the training sample with all candidate risk factors, we fit a logistic model with Markov Chain Monte Carlo (MCMC) simulation, and we calculated a posterior probability for each factor^14^. The posterior probability could assess the association strength between a factor and the outcome. Factors with a posterior probability > 0.95 (or < 0.05 for factors with estimates < 0.0) were considered significant for predicting the outcome and were included in the final risk factor list^15^. Then, we developed the final risk model to predict the outcome by fitting a logistic regression to the training sample data using the selected risk factors identified by the MCMC method.

We used the following indicators to evaluate the risk model performance: the Harrell C statistic to assess the overall predictive accuracy^16,17^, the McFadden R square to assess explained variation^18^, and the Hosmer–Lemeshow goodness-of-fit test to assess calibration^19^. Discrimination was assessed between the observed outcomes in strata defined by the predictive probabilities’ deciles. As described previously, we divided patients in the training sample into five mutually exclusive risk classes based on the deciles, ranking them from lowest (class 1) to highest risk (class 5) for evaluation^20^.

To further evaluate the selected risk factors, we used the training data to conduct a latent class analysis by an unsupervised machine learning algorithm that does not require a specified outcome^14^. Conceptually, unsupervised learning algorithms can assign patients to risk classes if the selected risk factors are substantially associated with the outcome. Thus, we used latent class analysis to classify patients into five mutually exclusive classes and ranked them from lowest (class 1) to highest risk (class 5) based on the observed outcome. We chose five classes to align with the decile-specific classes based on the risk model described previously. We calculated a Spearman correlation coefficient between the risk classes based on the risk model and the risk classes determined by the latent class analysis. A high coefficient was indicative of good agreement between the classified results, thereby providing information on the robustness of the selected risk factors.

We further validated the risk model by comparing its performance with the training sample, test sample and 2 independent validation samples.

#### Risk score

As described previously^15^, to facilitate the use of the selected risk factors and the risk model, we developed a simple risk score for each patient based on the regression coefficients estimated from the risk model with the training sample. Points for each risk factor were calculated by dividing the risk factor’s coefficient by the sum of all coefficients in the model, multiplying by 100, and rounding to the nearest integer. We stratified patients into 3 risk groups based on the distribution of the risk score: low (< 25th percentile), average (25th–75th percentile), and high (> 75th percentile).

All analyses were conducted using SAS^®^ software version 9.4 (SAS Institute Inc.). Latent class analysis was conducted using the PROC LCA method (version 1.3.2 beta). Nonlinear relationships were assessed using the PROC GAM method. This study followed the Transparent Reporting of a Multivariable Prediction Model for Individual Prognosis or Diagnosis (TRIPOD) reporting guideline. All 22 items of the TRIPOD statement were addressed.

## Results

### Study sample

A total of 1199 (609 training, 304 test, 207 validation by time, and 79 validation by site) participants were included in this study. The mean (SD) age was 35.1 (16.4) years, and 599 patients (50.0%) were female. There were some differences in the basic clinical characteristics of patients in training, test, and two validation samples. The differences between the training, test, and validation samples demonstrate the predictability and extrapolation of the model (Table 1).

**Table 1:** Patient characteristics by sample groups.

Characteristics	Aggregate	Training	Test	Validation_time	Validation_site	P_value
Total	1199	609	304	207	79	
Inhos_death n (%)	454 (37.9)	247 (40.6)	117 (38.5)	87 (42.0)	3 (3.8)	< 0.0001
Female n (%)	599 (50.0)	297 (48.8)	172 (56.6)	93 (44.9)	37 (46.8)	0.0444
Age Mean (std) median (q1, q3)	35.1 (16.4) 34 (23, 47)	34.7 (16.5) 34 (23, 46)	34 (16.2) 30.5 (23, 46)	36.1 (17.2) 34 (25, 48)	40.1 (12.8) 40 (29, 48)	0.0104
Age_lt_50 n (%)	963 (80.3)	495 (81.3)	246 (80.9)	159 (76.8)	63 (79.7)	0.5614
Age_ge_50 n (%)	236 (19.7)	114 (18.7)	58 (19.1)	48 (23.2)	16 (20.3)	0.5614
ALT1 Mean (std) median (q1, q3)	46.8 (114.6) 18 (12, 33)	46.8 (102.2) 18 (12, 34)	40.2 (81.1) 16 (12, 30.5)	62.1 (187.4) 19 (12, 41)	32 (32.5) 20 (13, 32)	0.4286
ALT1_lt_100	1073 (89.5)	546 (89.7)	274 (90.1)	179 (86.5)	74 (93.7)	0.3053
ALT1_ge_100	126 (10.5)	63 (10.3)	30 (9.9)	28 (13.5)	5 (6.3)	0.3053
AST1	57.1 (139) 23 (17, 39)	58.9 (146) 23 (18, 41)	53.3 (117.6) 23 (17, 37.5)	64.7 (168.3) 23.2 (18, 41)	38.3 (39.3) 22 (17, 32)	0.3409
AST1_lt_60	982 (81.9)	498 (81.8)	249 (81.9)	170 (82.1)	65 (82.3)	0.9992
AST1_ge_60	217 (18.1)	111 (18.2)	55 (18.1)	37 (17.9)	14 (17.7)	0.9992
BUN1	6.8 (5.8) 5.2 (4, 7.4)	6.8 (5.5) 5.4 (4, 7.7)	6.7 (5.5) 5 (3.9, 7.3)	7.1 (7.2) 5.2 (4, 7.3)	6.1 (5.4) 4.9 (4.1, 6.5)	0.3161
BUN1_lt_10	1045 (87.2)	530 (87.0)	259 (85.2)	180 (87.0)	76 (96.2)	0.0774
BUN1_ge_10	154 (12.8)	79 (13.0)	45 (14.8)	27 (13.0)	3 (3.8)	0.0774
CK_MB1	29.4 (38.6) 20.2 (12.9, 33.6)	31 (39.6) 21 (13.6, 36)	31.3 (40.3) 20.9 (13.8, 32.7)	28.6 (37.7) 19.1 (12.3, 33.3)	12.1 (14) 3.8 (2, 25.5)	< 0.0001
CK_MB1_lt_50	1054 (87.9)	518 (85.1)	269 (88.5)	189 (91.3)	78 (98.7)	0.0013
CK_MB1_ge_50	145 (12.1)	91 (14.9)	35 (11.5)	18 (8.7)	1 (1.3)	0.0013
CR_1	108.4 (124.7) 66 (51, 117)	108 (108.2) 67 (51, 123)	109 (123.1) 66 (49.5, 117)	114.4 (152.9) 66 (52, 105.5)	92.6 (163.1) 60.7 (51.4, 81.6)	0.4877
CR_1_lt_150	985 (82.2)	492 (80.8)	251 (82.6)	168 (81.2)	74 (93.7)	0.0441
CR_1_ge_150	214 (17.8)	117 (19.2)	53 (17.4)	39 (18.8)	5 (6.3)	0.0441
DBiL1	10.2 (22.3) 5.1 (3.4, 7.7)	11 (23.5) 5.1 (3.6, 7.8)	9.1 (14.1) 5.4 (3.5, 8.1)	11.2 (30.7) 4.8 (3.3, 6.8)	6.4 (6.3) 4 (2.9, 6.1)	0.0033
DBiL1_lt_20	1080 (90.1)	541 (88.8)	277 (91.1)	188 (90.8)	74 (93.7)	0.4418
DBiL1_ge_20	119 (9.9)	68 (11.2)	27 (8.9)	19 (9.2)	5 (6.3)	0.4418
GGT1	50.7 (95.8) 21 (14, 47.8)	50.6 (101.3) 19 (13.4, 37.3)	43.4 (74.1) 18.5 (13.4, 40)	56 (119.1) 23.9 (14, 50)	65.6 (42.1) 61.4 (47, 79.2)	< 0.0001
GGT1_lt_150	1134 (94.6)	569 (93.4)	291 (95.7)	196 (94.7)	78 (98.7)	0.1714
GGT1_ge_150	65 (5.4)	40 (6.6)	13 (4.3)	11 (5.3)	1 (1.3)	0.1714
MPV	9.2 (3.6) 8.9 (8, 9.9)	9.1 (1.5) 9 (8.1, 9.8)	9.2 (1.6) 8.9 (8, 10.2)	9.6 (8.1) 8.6 (7.9, 9.7)	9.2 (1.1) 9.2 (8.3, 9.9)	0.0487
MPV_lt_10d5	1004 (83.7)	518 (85.1)	238 (78.3)	179 (86.5)	69 (87.3)	0.0256
MPV_ge_10d5	195 (16.3)	91 (14.9)	66 (21.7)	28 (13.5)	10 (12.7)	0.0256
N1	83.8 (13.4) 88.9 (79.3, 92.5)	84.3 (13.2) 89.2 (80.2, 92.6)	84.3 (13.8) 89.5 (81.1, 92.6)	82.7 (13.7) 87.9 (76.2, 92.7)	81 (11.1) 84.1 (74.2, 89)	0.0007
N1_lt_80	310 (25.9)	149 (24.5)	68 (22.4)	60 (29.0)	33 (41.8)	0.0029
N1_ge_80	889 (74.1)	460 (75.5)	236 (77.6)	147 (71.0)	46 (58.2)	0.0029
PCT	0.2 (0.1) 0.2 (0.1, 0.2)	0.1 (0.1) 0.1 (0.1, 0.2)	0.1 (0.1) 0.1 (0.1, 0.2)	0.2 (0.1) 0.2 (0.1, 0.2)	0.1 (0.1) 0.1 (0.1, 0.2)	< 0.0001
PCT_lt_0d2	903 (75.3)	483 (79.3)	240 (78.9)	119 (57.5)	61 (77.2)	< 0.0001
PCT_ge_0d2	296 (24.7)	126 (20.7)	64 (21.1)	88 (42.5)	18 (22.8)	< 0.0001
PLT	179.4 (90) 172 (113, 236)	173.6 (87.7) 165 (109, 226)	170.2 (90.5) 157 (107, 218)	218.4 (88) 211 (167, 271)	158.1 (84) 161 (80, 220)	< 0.0001
PLT_lt_80	155 (12.9)	79 (13.0)	49 (16.1)	11 (5.3)	16 (20.3)	0.0006
PLT_ge_80	1044 (87.1)	530 (87.0)	255 (83.9)	196 (94.7)	63 (79.7)	0.0006
WBC1	14.1 (8) 11.8 (8.6, 17.3)	14.4 (8.4) 11.9 (8.6, 18.5)	14.4 (7.9) 12.4 (9.2, 17.7)	13.2 (7.1) 11.1 (8.3, 16.5)	12.3 (6.5) 10.6 (8.3, 14.4)	0.0373
WBC1_lt_20	979 (81.7)	484 (79.5)	250 (82.2)	173 (83.6)	72 (91.1)	0.0642
WBC1_ge_20	220 (18.3)	125 (20.5)	54 (17.8)	34 (16.4)	7 (8.9)	0.0642
	279 (151)	289.4 (102.8)	310.8 (165.4)	356.2 (253.3)	219.3 (58.1)	
LDH1	236.1 (205, 299.3)	273.3 (218.4, 333)	259.7 (235.5, 320.5)	265.1 (221.7, 363.3)	209.6 (182.8, 234.8)	< 0.0001
LDH1_lt_240	1101 (91.8)	579 (95.1)	277 (91.1)	184 (88.9)	61 (77.2)	< 0.0001
LDH1_ge_240	98 (8.2)	30 (4.9)	27 (8.9)	23 (11.1)	18 (22.8)	< 0.0001
Ingestion_volume	61.1 (67.5) 40 (20, 80)	65.1 (69.9) 40 (20, 100)	55.8 (63.4) 30 (15, 80)	61.5 (72) 30 (15, 80)	49.8 (47.3) 40 (20, 60)	0.2922
Ingestion_volume_lt_100	927 (77.3)	452 (74.2)	244 (80.3)	161 (77.8)	70 (88.6)	0.0141
Ingestion_volume_ge_100	272 (22.7)	157 (25.8)	60 (19.7)	46 (22.2)	9 (11.4)	0.0141
LOS	7.3 (6.8) 5 (2, 11)	7.4 (7) 5 (2, 10)	7.9 (6.9) 6 (2, 11.5)	6.9 (6.1) 5.1 (2, 10.7)	5.5 (5.8) 3 (1, 8)	0.0045

*MPV* mean platelet volume, *PCT* procalcitonin, *GGT* gamma-glutamyl transferase, *CR* creatinine.

### In-hospital death events

In-hospital death rates were 40.6% (95% CI 36.6–44.6%) and 38.5% (95% CI 33.0–44.2%) for the training and test samples. The average in-hospital death rate was 37.9%. The median (interquartile range [IQR]) hospital stay was 5 (2–11) days (Table 1).

### Risk factor selection and testing

**Figure 2:**
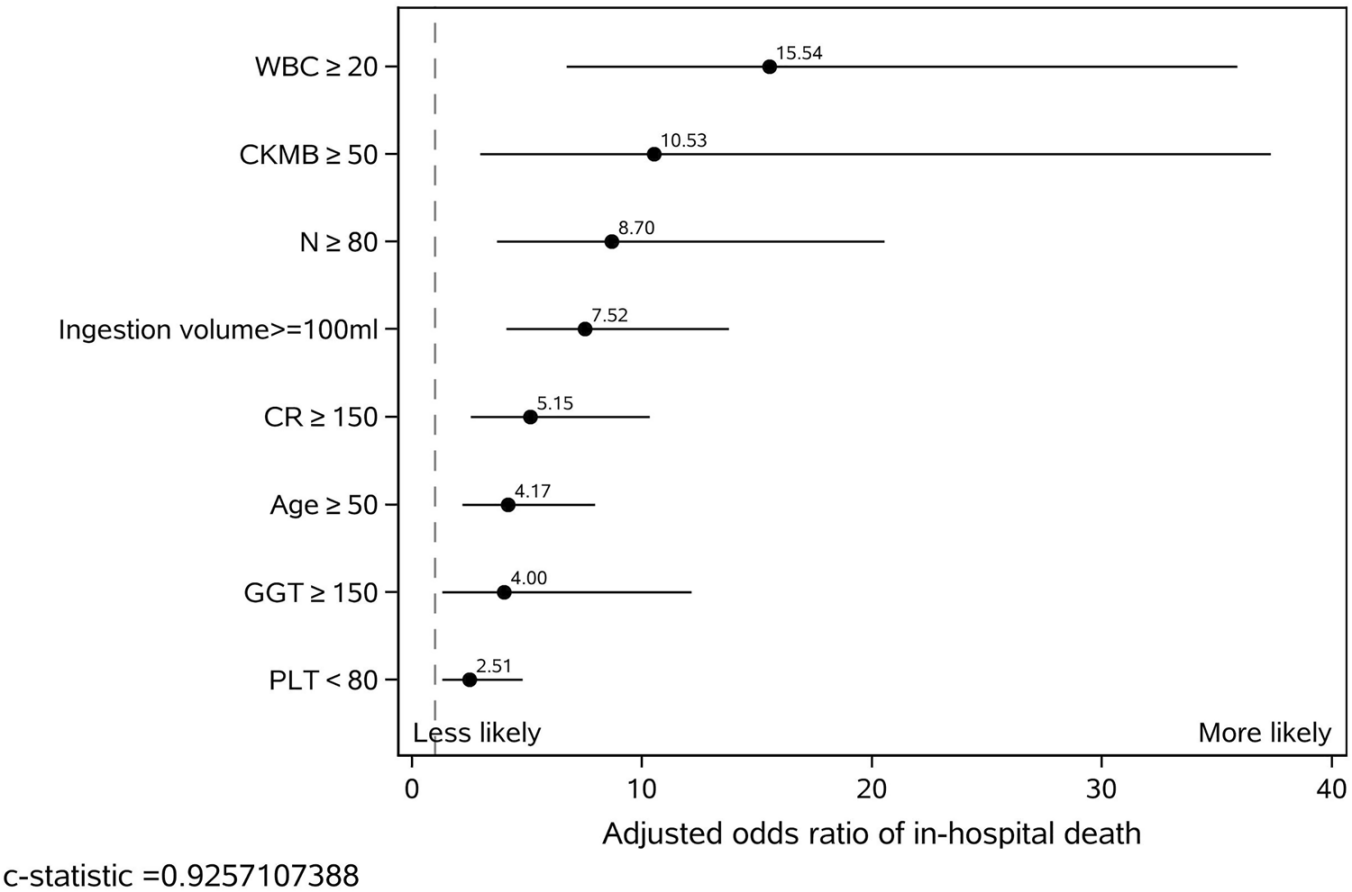
Risk factors associated with in-hospital death based on the training sample. Eight candidate factors, including age, ingestion volume, CK-MB, PLT, WBC, N, GGT, and Cr, were identified using the MCMC simulation. Factors with a posterior probability > 0.95 were considered significant for predicting the outcome.

**Figure 3:**
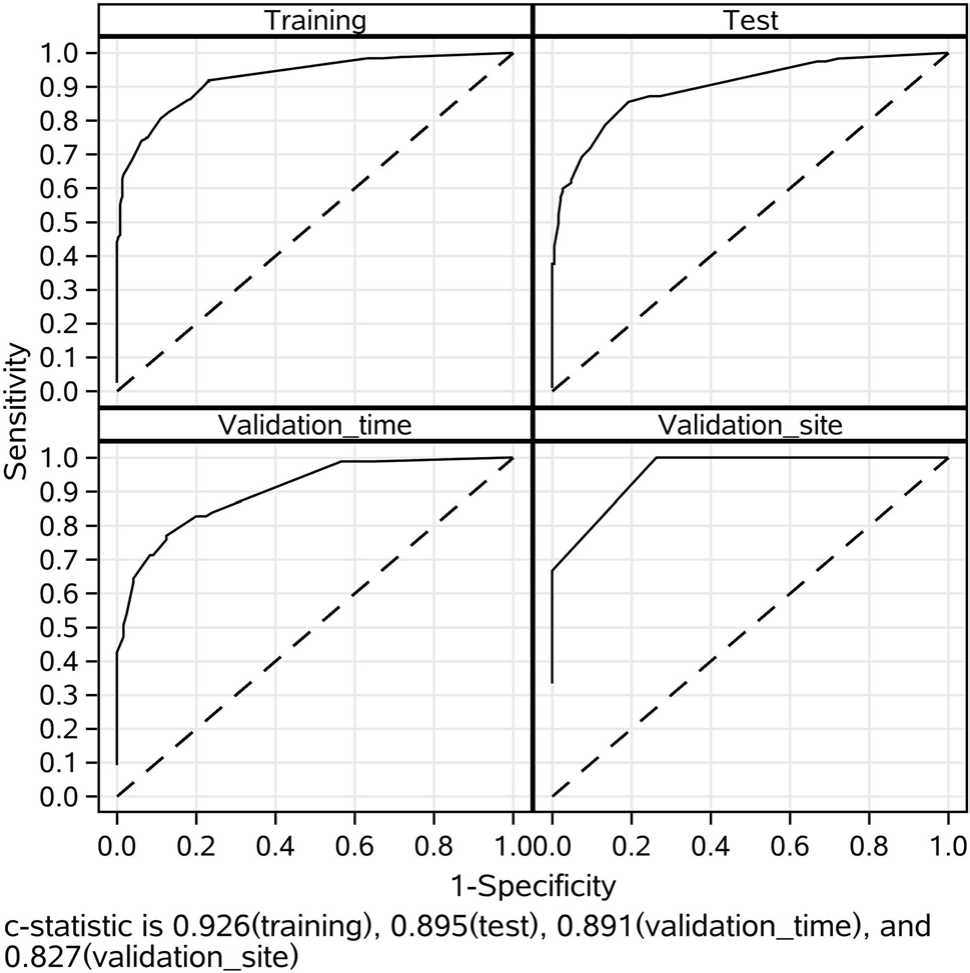
ROC analyses for the evaluation and validation of the risk model in the training, test, validation_time, and validation_site samples. Model performance in the test, validation_time, and validation_site samples was comparable to that in the training sample. The overall C statistics were 0.926 (95% CI 0.891–0.924), 0.895 (95% CI 0.855–0.928), 0.891 (95% CI 0.848–0.932), and 0.827 (95% CI 0.455–1.000) for the training, test, validation_time, and validation_site samples, respectively.

**Figure 4:**
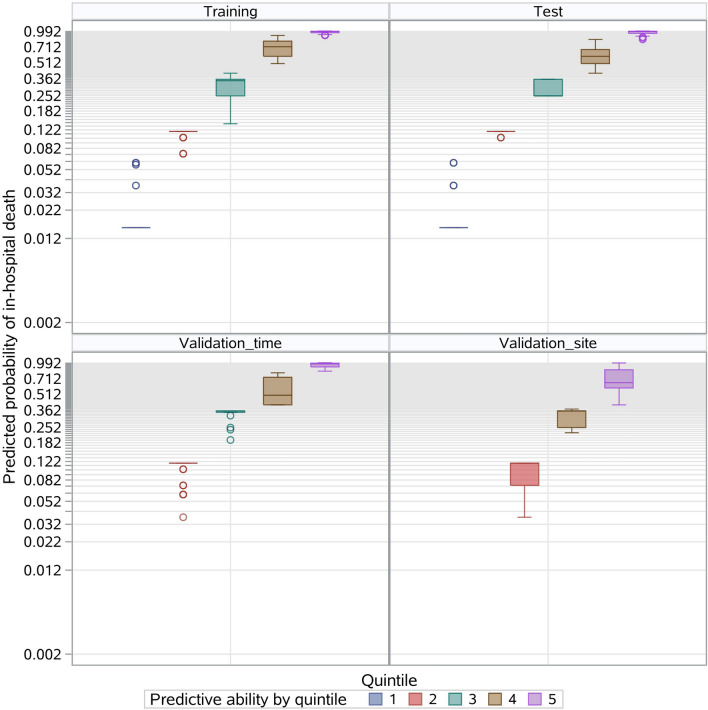
Probability of in-hospital death events by quintiles in the training, test, and validation_time and validation_site samples. Patients in each sample were divided into five classes based on the combination of eight risk factors.

**Figure 5:**
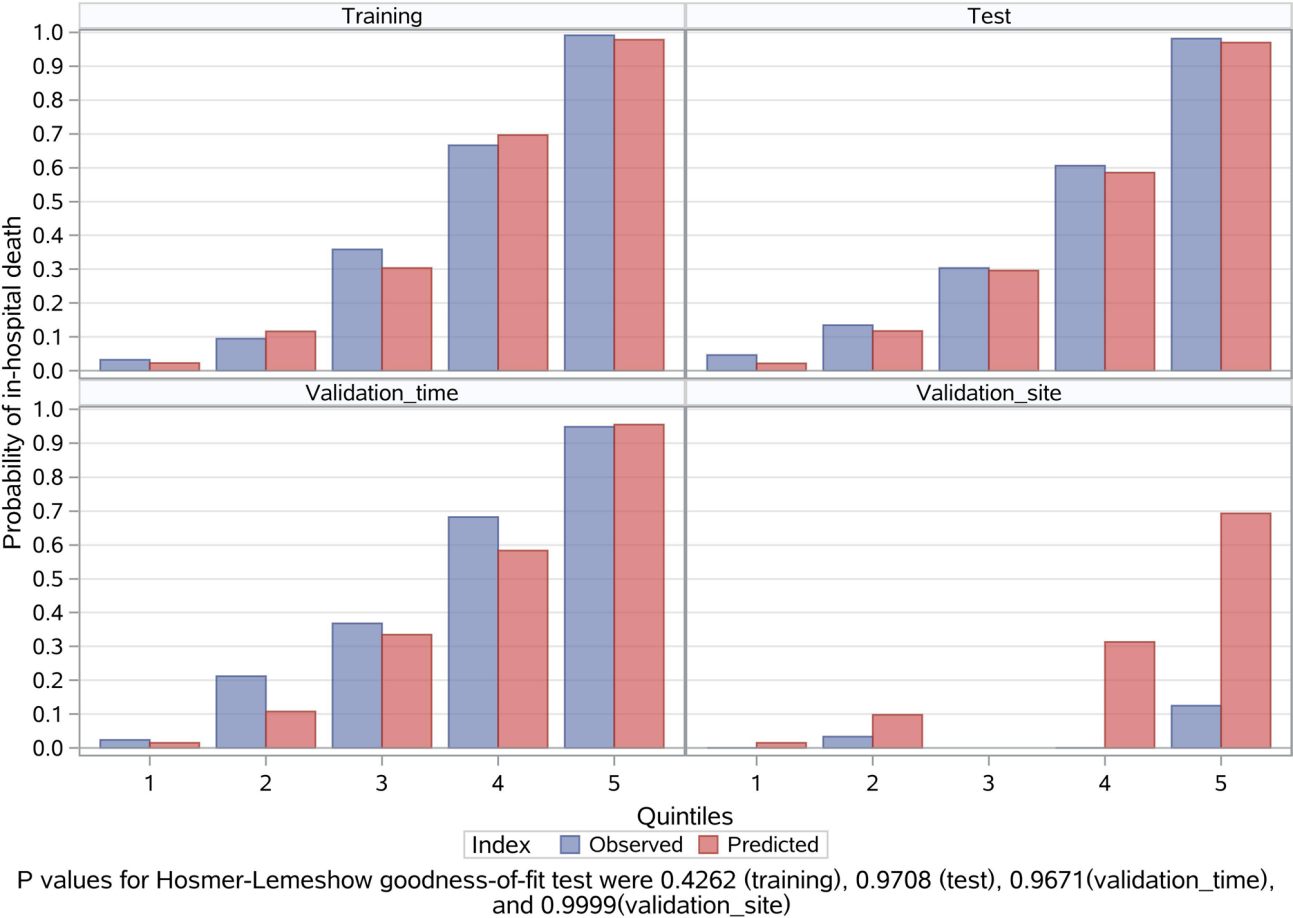
Observed versus predicted values by quintiles in the training, test, validation_time, and validation_site groups. The Hosmer and Lemeshow’s Goodness of Fit Test’ p-values were 0.4262, 0.9708, 0.9671, and 0.9999 for the training, test, validation_time, and validation_site samples, respectively.

The MCMC simulation identified eight candidate factors with a posterior probability of at least 0.95 (Table 2), including age, ingestion volume, CK-MB, PLT, WBC, N, GGT, and Cr (Fig. 2). The risk model based on the eight risk factors and the training sample demonstrated good discrimination, calibration, and fit. The overall C statistic was 0.926 (95% CI 0.891–0.924) for the risk model (Fig. 3). The mean observed in-hospital death rate ranged from 3.3% in the lowest predicted quintile to 99.2% in the highest predicted quintile, with a range of 95.9% and variation of 0.4999 (Fig. 4). Moreover, the P-values of the Hosmer–Lemeshow goodness-of-fit test were 0.4262 in the training sample and 0.9078 in the test sample, indicating a good fit with the test cohort (Fig. 5).

**Table 2:** Final risk prediction model for in-hospital death based on training sample.

Variable	Estimate	Std Err	Score_R^a^
WBC1 > 20	2.743299958	0.42703288	19
CK_MB1 > 50	2.35461446	0.6455247	16
N1 > 80	2.162847223	0.438185092	15
Ingestion_volume > 100	2.018090939	0.3082356	14
CR_1 > 150	1.638231401	0.356155153	11
Age > 50	1.427619459	0.329849932	10
GGT1 > 150	1.387451403	0.566479088	9
PLT < 80	0.921076865	0.331850564	6
Total			100

^a^Risk scores were calculated by dividing a risk factor's coefficient by the sum of all coefficients, multiplying by 100, and rounding to the nearest integer.

Additionally, model performance with the test sample was comparable to that with the training sample. The overall C Statistic was 0.895 (95% CI 0.855–0.928) in the test sample (Fig. 3). The observed in-hospital death rate ranged from 4.6% in the lowest predicted quintile to 98.2% in the highest predicted quintile, and the explained variation was 0.4182 for the test sample (Fig. 4).

**Figure 6:**
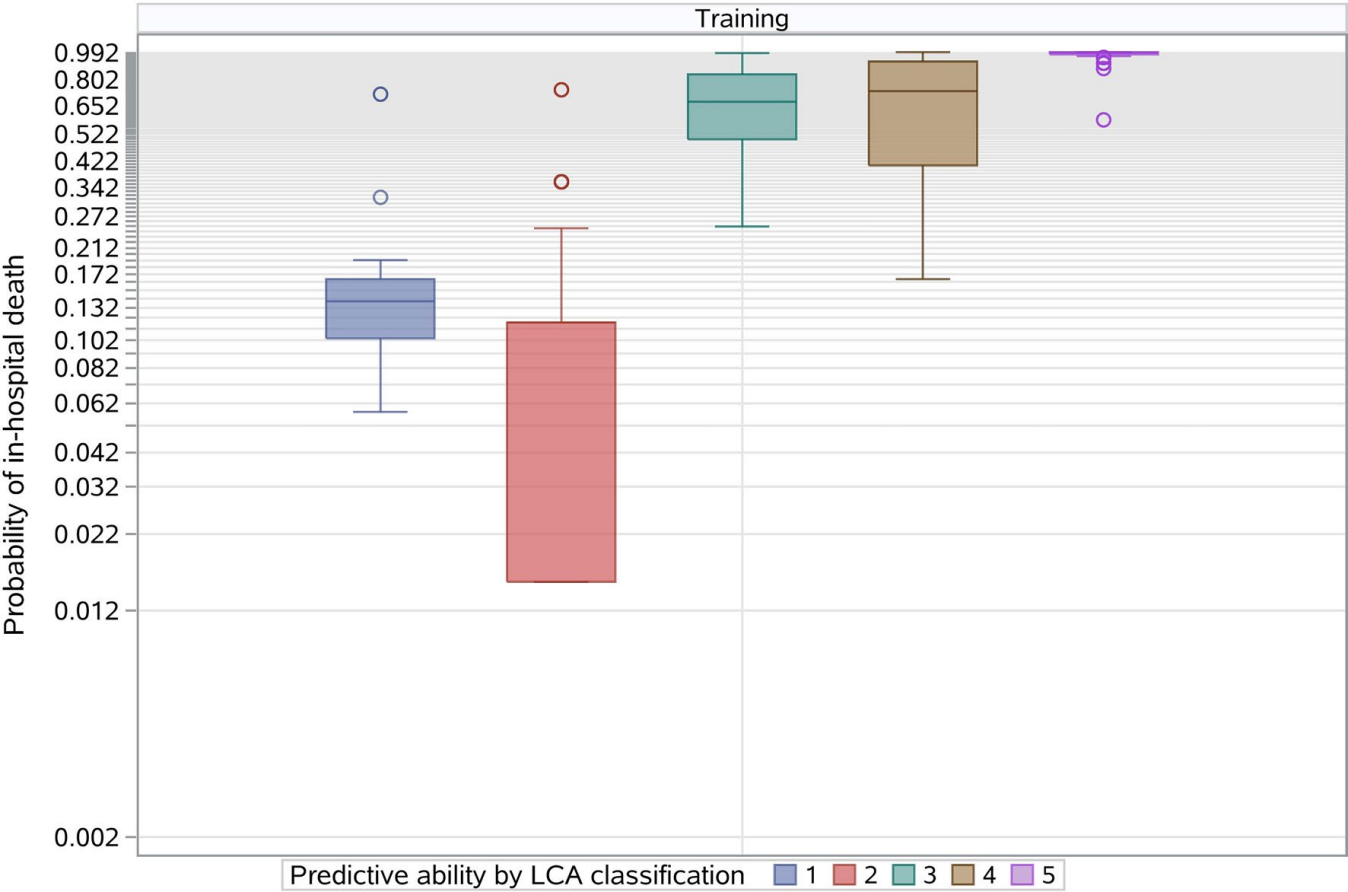
Risk stratification based on latent class analysis in the training sample. A total of 609 patients in the training sample were divided into five classes based on the eight risk factors. The mean observed outcome rate ranged from 0.0% in the lowest rating class to 99.2% in the highest rating class.

Furthermore, in the latent class analysis, 609 patients in the training sample were assigned into five classes based on the combination of the eight risk factors (Fig. 6). For this analysis, the area under the receiver operating curve (ROC) curve was 0.877 (95% CI 0.832–0.906), and the mean observed in-hospital death rate ranged from 0.0% in the lowest- to 99.2% in the highest rating group. The Spearman’s correlation coefficient between the predicted quintile based on the logistic model and the latent class analysis was 0.754 (95% CI 0.715–0.874).

### Risk score system

**Figure 7:**
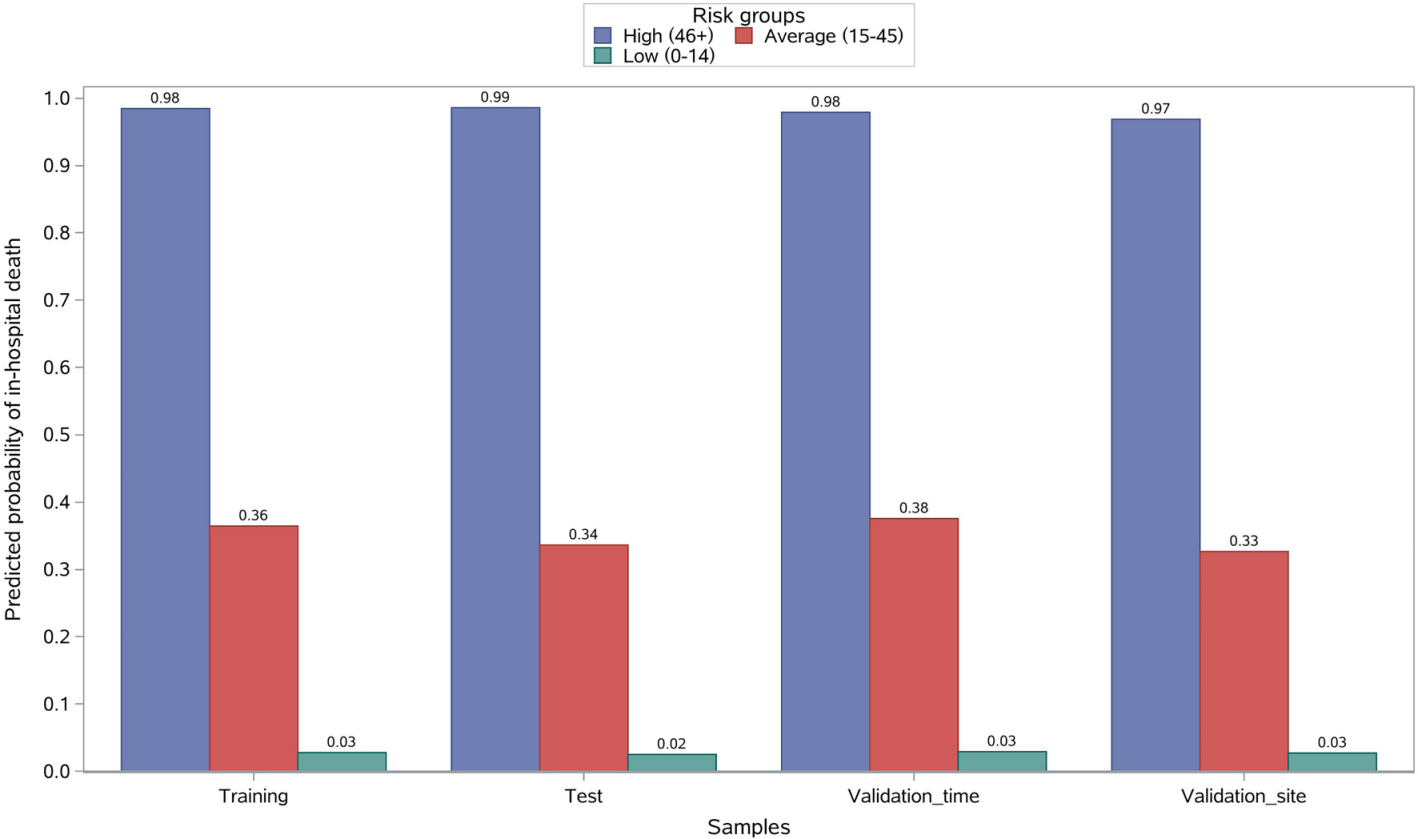
Risk stratification by risk scores. For the training, test, validation_time, and validation_site samples, respectively, the predicted probabilities of in-hospital death for PQ poisoning patients were 98%, 99%, 98%, and 97% in the highest risk group (46+), 59.9%, 62.2%, 59.4%, and 60.8% in the average-risk group (15–45), and 21.7%, 22.4%, 27.1%, and 36.7% in the lowest risk group (0–14).

The risk factor-specific points ranged from 19 (WBC ≥ 20) to 6 (PLT < 80) (Table 2). WBC ≥ 20, CK-MB ≥ 50, N ≥ 80, ingestion volume ≥ 100, and Cr ≥ 150 were the top five factors with odds ratios > 5.0 (Fig. 2). The training sample had a mean (SD) risk score of 26.6 (21.6). The mean (SD) score of the test sample was 24.8 (21.1). Further, in the training sample, 18.4%, 59.9%, and 21.7% of patients were stratified into the high, average, and low-risk groups, with corresponding probabilities of 0.985, 0.365, and 0.03 for in-hospital death, respectively (Fig. 7). The stratification of the test sample was not markedly different from that of the training sample (Fig. 7, Table 3).

**Table 3:** Patient risk stratification based on risk score.

Risk group	Training (n = 609)		Test (n = 304)		Validation_time (n = 207)		Validation_site (n = 79)	
	Patients # (%)	Probability of in-hospital death, mean (std)	Patients # (%)	Probability of in-hospital death, mean (std)	Patients # (%)	Probability of in-hospital death, mean (std)	Patients # (%)	Probability of in-hospital death, mean (std)
High (46+)	112 (18.4)	0.98 (0.02)	47 (15.5)	0.99 (0.02)	28 (13.5)	0.98 (0.02)	2 (2.5)	0.97 (0.04)
Average (15–45)	365 (59.9)	0.36 (0.28)	189 (62.2)	0.34 (0.25)	123(59.4)	0.38 (0.27)	48 (60.8)	0.33 (0.24)
Low (0–14)	132 (21.7)	0.03 (0.02)	68 (22.4)	0.02 (0.02)	56 (27.1)	0.03 (0.03)	29 (36.7)	0.03 (0.02)

### Risk model validation

The in-hospital death events rates were 42.0% (95% CI 35.2–49.1%) and 3.8% (95% CI 0.8–10.7%) for the time and site validation samples, respectively (Table 1). The observed in-hospital death rates ranged from 2.3% and 0.0% in the lowest predicted quintiles to 94.9% and 12.5% in the highest predictive quintiles, and the explained variations were 0.3798 and 0.2421 for the time and site validation samples, respectively. The overall C statistics were 0.891 (95% CI 0.848–0.932) and 0.827 (95% CI 0.455–1.000) (Fig. 3), and the P-values of the Hosmer and Lemeshow's Goodness of Fit Test were 0.9671 and 0.9999 for the two independents samples (Fig. 5).

The mean (SD) risk scores were 23.5 (19.7) and 16.3 (14.1) for the time and site validation samples, respectively. In the validation_time sample, 27.1%, 59.4%, and 13.5% of patients were classified into the low-, average-, and high-risk groups, respectively, with corresponding probabilities of 0.98, 0.38, and 0.03 for in-hospital death events (Fig. 7 and Table 3). In the validation_site group, 36.7%, 60.8%, and 2.5% of patients were classified into the low-, average-, and high-risk groups, with corresponding probabilities of in-hospital death events of 0.03, 0.33, and 0.97. The probabilities for in-hospital death events of the validation samples were identical to those of the training sample (Fig. 7 and Table 3).

## Discussion

In this study, we evaluated 609 patients with acute PQ poisoning, and we identified eight prognostic factors for survival after PQ poisoning. Furthermore, a random forest risk model and a scoring system based on these risk factors were developed to predict the in-hospital death probability for patients diagnosed with PQ poisoning. Importantly, our model was tested both in internal and external validation samples. The risk factors were selected based on data from medical records and ease of collection and availability after discharge and during long-term follow-up. Furthermore, the statistical algorithms used in this study are robust. The risk prediction model and its corresponding risk scoring system may help clinicians to identify the patients at highest risk of in-hospital death after PQ poisoning.

PQ exposure is a major cause of fatal poisoning in most Asian nations. PQ can cause severe multiple organ damage, such as lungs and gastrointestinal tract, which are the main causes of death after PQ poisoning^21–23^. Although therapeutic strategies for the management of acute PQ poisoning have been extensively investigated, PQ poisoning remains poor^24,25^. Generally, the severity and prognosis of acute poisoning are determined by the ingested dose. Previous studies have reported that plasma PQ concentrations can be used to predict clinical outcomes for patients with PQ poisoning^26,27^. In view of plasma PQ concentration is quantitatively measured using radioimmunoassay or liquid chromatography-mass spectrometry methods, which cannot be done in most hospitals, we used PQ ingestion volume rather than PQ plasma concentration at present medical situation. Moreover, Patients with PQ poisoning were conscious on admission, including critical PQ poisoning patients. Clinicians can easily obtain PQ ingestion volume data from the patients or their relatives. Identifying potential risk factors to predict the clinical outcome of PQ poisoning patients at an early stage may improve early diagnosis and facilitate rapid medical intervention for patients who have the highest risk of in-hospital death. Therefore, there is an urgent need to develop a reliable and universal risk prediction model based on available laboratory data to evaluate the early prognosis of PQ poisoning patients. Furthermore, validating and classifying the risk of in-hospital death based on a multi-clinical factor index model is an important innovation in emergency medical research, which could aid in risk stratification and therapeutic regimen adjustment for patients with acute PQ poisoning.

The MCMC algorithm was used to evaluate the association strength between the risk factors and the outcome. Compared with prior studies, the clinical indicators included in this model were routine and inexpensive, and every hospital could easily acquire the data. Our model had better predictive accuracy based on two external validation samples from a different time and site, which were used to validate our scoring system. The SIPP has been recognized as a valuable prognostic model for PQ poisoning patients in previous studies^9,28^. The unavailability of SIPP indicators in many hospitals makes it difficult to apply the scoring system routinely, limiting the accurate evaluation of PQ poisoning severity. The APACHE II scoring system is also in clinical settings to evaluate the prognosis of PQ poisoning patients^29,30^. However, this scoring system might underestimate mortality in critically ill patients. Our prediction model was developed using the baseline clinical indicators of PQ poisoning patients. It is easy to use and allows the exclusion of terminally-ill or minimally affected patients from needless aggressive therapy.

Hidden factors used for risk model development may affect the efficiency of the model in different contexts. Effective risk prediction factors must be supported by clinical research, and the data must be easily collected and available during hospitalization as well as after discharge. The risk factors in our study were tested and validated and had good agreement with the training results. Notably, the eight risk factors selected for this study meet all the criteria listed above. Our risk model includes age, ingestion volume, CK-MB, PLT, WBC, N, GGT, and Cr. Previous studies demonstrated that WBC and neutrophil counts were essential indexes for the prognosis of PQ poisoning patients^31,32^. Creatinine, one of the risk factors associated with a poor prognosis, could be induced by direct oxidative injury in renal tubules^33^. Elevated serum Cr level is closely associated with acute kidney injury (AKI). Further, PQ poisoning patients with AKI had a higher mortality risk than those with normal kidney function^34^. Through risk stratification, we found that in the training sample, the proportion of patients at high risk or moderate risk of in-hospital death after PQ poisoning was 98% and 36%, respectively. The eight risk factors we identified could help clinicians make medical interventions that may improve the prognosis and reduce unnecessary treatment of PQ poisoning patients. Improving the clinical outcomes of PQ poisoning patients and reducing in-hospital death rates would decrease the economic burden on the healthcare system.

## Conclusion

The early clinical outcomes for PQ poisoning patients have been difficult to evaluate. The novel risk model we developed is easy to use and can exclude the terminally-ill or minimally affected PQ poisoning patients from needless aggressive therapy better than conventional scoring systems such as APACHE II, SOFA, and SIPP. This risk score system is suitable for clinical use for recognizing high-risk PQ poisoning patients and for predicting the probability of in-hospital death.

## Supplementary Information

Supplementary Information.
