# Supplementary material for: A novel simple risk model to predict the prognosis of patients with paraquat poisoning
Source: Sci Rep. 2021 Jan 8;11:237. doi: 10.1038/s41598-020-80371-5 (PMC7794476; doi:10.1038/s41598-020-80371-5)

Title page:

A novel simple risk model to predict the prognosis of patients with paraquat poisoning

Short titles: A simple risk model to predict paraquat poisoning prognosis

Yanxia Gao[a†], Liwen Liu[b, c†], Tiegang Li[d†], Ding Yuan[a], Yibo Wang[a], Zhigao Xu[a], Linlin Hou[a], Yan Zhang[a], Guoyu Duan[a], Changhua Sun[a], Lu Che[a], Sujuan Li[a], Pei Sun[a], Yi Li[e*], Zhigang Ren[b, c*]

Table S1. The distribution information of variables in the 1199 samples.

variable	mean(std)	median (IQR)	min	max	missing number	missing rate	tag
age	35.2 (16.4)	34.0 (24.0-47.0)	1.0	90.0	13	1.1	impute
LOS	7.3 (6.9)	5.0 (2.0-11.0)	1.0	42.0	38	3.1	impute
ingestion_volume	60.6 (69.8)	30.0 (15.0-80.0)	0.5	500.0	105	8.6	impute
N1	83.8 (13.6)	89.1 (79.4-92.5)	5.1	98.3	75	6.1	impute
WBC1	14.0 (8.1)	11.8 (8.5-17.0)	2.8	93.0	77	6.3	impute
AST1	52.5 (141.3)	23.0 (17.0-34.0)	1.0	2025.0	84	6.9	impute
ALT1	42.8 (117.0)	17.0 (12.0-29.0)	2.0	2336.0	90	7.4	impute
GGT1	47.0 (100.7)	18.5 (13.3-33.2)	2.0	1274.0	146	11.9	impute
CK_MB1	27.6 (39.7)	17.8 (12.3-30.0)	0.6	617.6	162	13.3	impute
BUN1	6.8 (7.7)	5.0 (3.9-7.0)	1.4	178.0	95	7.8	impute
CR_1	106.1(128.5)	63.6 (50.0-102.0)	17.0	1633.0	94	7.7	impute
MPV	9.2 (3.7)	8.9 (8.0-9.9)	0.8	90.8	77	6.3	impute
PLT	179.3 (92.0)	171.0 (110.0-240.0)	0.1	641.0	77	6.3	impute
PCT	0.2 (0.1)	0.2 (0.1-0.2)	0.0	0.6	83	6.8	impute
LDH1	273.6(194.1)	217.0 (177.0-294.0)	61.0	2224.0	148	12.9	impute
DBiL1	9.4 (22.8)	4.9 (3.3-6.7)	0.4	288.4	98	8.0	impute
CK1	281.1(1287.2)	111.0 (72.0-182.5)	0.0	32034.0	214	17.5	delete
PaCO2_1	31.6 (8.4)	31.9 (26.3-36.7)	7.4	77.0	668	54.7	delete
Amylase_1	228.6 (425.9)	93.0 (55.0-198.0)	4.0	3557.0	425	34.8	delete
Lipase_1	139.0 (441.8)	24.3 (17.2-44.0)	1.6	6222.0	459	37.6	delete
PaCO2	31.5 (8.4)	31.9 (26.2-36.6)	7.4	77.0	656	53.7	delete
PQ_concentration	151.4 (877.7)	3.3 (0.2-46.6)	0.0	24864.0	240	19.6	delete
CD3	651.8 (664.3)	449.5 (301.0-785.0)	63.0	4944.0	1074	87.9	delete
CD4	331.8 (348.1)	224.0 (144.0-370.0)	32.0	2544.0	994	87.0	delete
CD8	282.3 (307.0)	196.0 (128.0-331.0)	24.0	2304.0	994	87.0	delete
IgM1	1.2 (0.6)	1.0 (0.8-1.2)	0.5	3.1	1112	97.3	delete
IgA1	1.1 (0.6)	0.9 (0.7-1.5)	0.4	2.5	1112	97.3	delete
IgG1	8.3 (2.4)	8.6 (6.5-9.4)	4.8	17.4	1112	97.3	delete
C3_1	1.0 (0.2)	1.0 (0.9-1.1)	0.5	1.7	1110	97.1	delete
C4_1	0.2 (0.1)	0.2 (0.2-0.3)	0.1	0.8	1110	97.1	delete
TBiL1	17.1 (20.0)	12.3 (8.4-17.9)	0.6	304.9	288	23.6	delete
cTnI1	0.0 (0.2)	0.0 (0.0-0.0)	0.0	1.8	951	77.8	delete
cystain_C	7.3 (3.7)	8.4 (6.6-9.6)	0.3	15.6	525	43.0	delete
PH_1	7.4 (0.2)	7.4 (7.3-7.4)	6.8	9.6	681	55.7	delete
Pao2_1	83.6 (27.3)	84.0 (65.3-101.0)	11.0	188.0	682	55.8	delete
LAC1	4.1 (4.0)	2.6 (1.5-4.9)	0.3	26.0	700	57.3	delete
HCO3_1	18.7 (6.2)	19.7 (14.2-23.1)	2.1	41.8	700	57.3	delete

Figure S1. The relationship of in-hospital death and variables

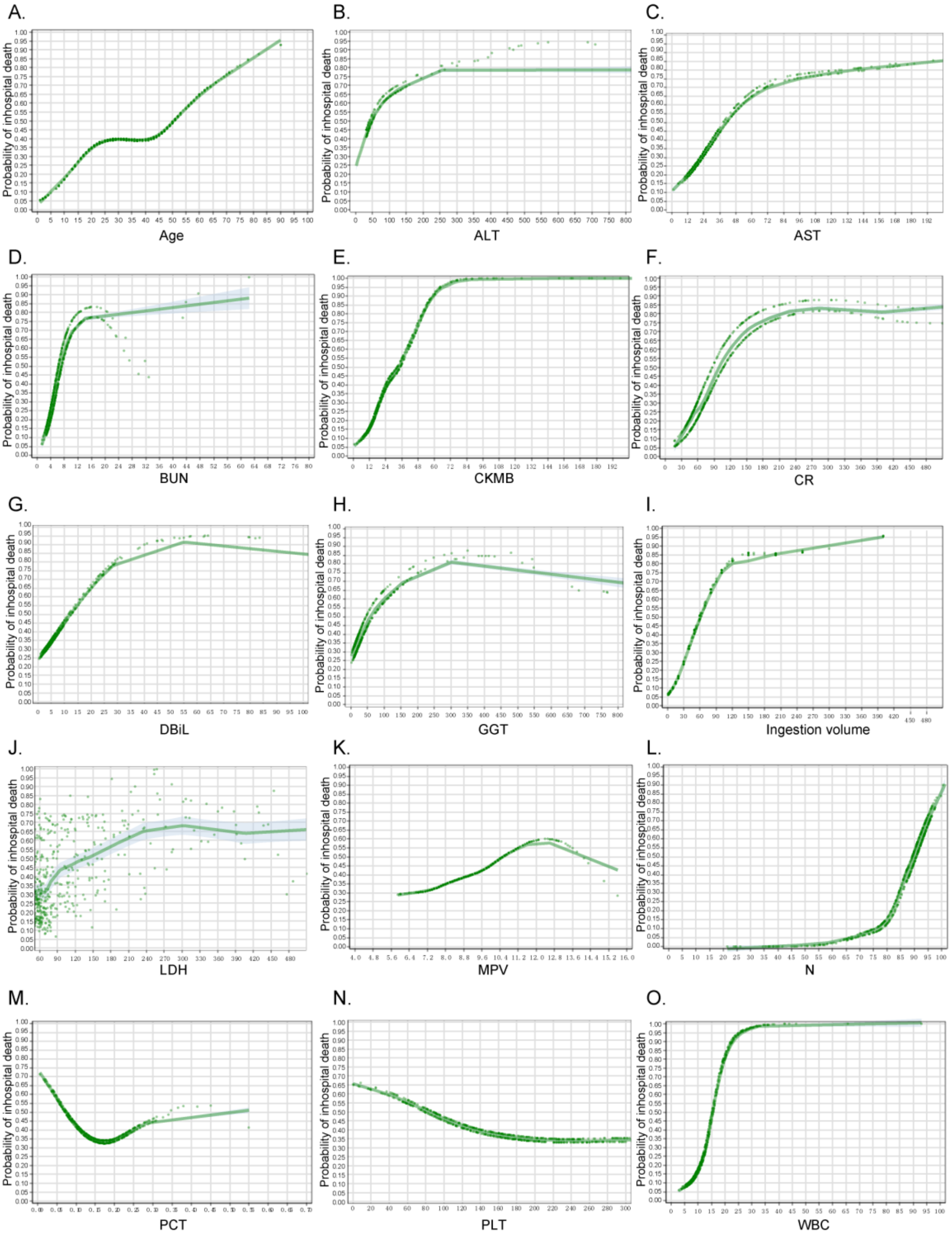

Supplement: Supplementary file 1 — Supplementary Information. [file 41598_2020_80371_MOESM1_ESM.pdf]
